# Summary of 2185 prenatal invasive procedures in a single center: A retrospective analysis

**DOI:** 10.4274/tjod.36097

**Published:** 2017-06-15

**Authors:** Hüseyin Çağlayan Özcan, Mete Gürol Uğur, Seyhun Sucu, Aynur Mustafa, Neslihan Bayramoğlu Tepe, Özcan Balat

**Affiliations:** 1 Gaziantep University Faculty of Medicine, Department of Obstetrics and Gynecology, Gaziantep, Turkey

**Keywords:** Amniocentesis, chorionic villus sampling, cordocenteses, prenatal genetic diagnostic testing

## Abstract

**Objective:**

To determine the frequency, indications, and outcomes of diagnostic invasive prenatal procedures (DIPP) performed in a university hospital.

**Materials and Methods:**

This retrospective, observational study included 2185 cases of DIPP (chorionic villus sampling, amniocentesis, and cordocentesis) performed at the department of obstetrics and gynecology of a university hospital between 2010 and 2016. We included all DIPP cases performed between 11 and 24 weeks of gestation. We compared the different types of DIPP performed in our hospital.

**Results:**

Two thousand one hundred eighty-five procedures were performed (1853 amniocenteses, 326 chorionic villus sampling, and 6 cordocenteses). The main indication for performing invasive procedures was abnormal results of aneuploidy screening for trisomy 21, followed by maternal age, and fetal structural abnormality. The fetal karyotype was altered in 154 (26.1%) cases. Trisomy 21 was the most common aneuploidy followed by trisomy 18, monosomy X, and trisomy 13. Fetal karyotype could not be revealed in 42 (2%) cases due to maternal contamination in 18 cases, inadequate sampling in 4 cases, and failure of cell culture in 27 cases. There were 2 pregnancy losses due to the invasive procedure (only in amniocentesis).

**Conclusion:**

The ideal approach to pregnancies with a detected chromosomal abnormality should be tailored according to the individual choice of the couples regarding whether they decide for or against a child with a known chromosomal abnormality.

## PRECIS:

The ideal approach to pregnancies with a chromosomal abnormality should be tailored according to the individual choice of the couples regarding whether they decide for or against a child with a known chromosomal abnormality.

## INTRODUCTION

Prenatal genetic diagnostic testing is intended to determine if a specific genetic disorder or condition is present in the fetus with as much certainty as possible. In contrast, prenatal genetic screening is designed to assess whether a patient is at increased risk of having a fetus affected by a genetic disorder. Physicians use various methods to determine high-risk pregnancies that are candidates for prenatal detection of chromosomal abnormalities (CA) with invasive diagnostic procedures. Autosomal aneuploidy (especially trisomy 21, 18, and 13) screening is the most common and cost-effective prenatal screening test. Currently, first-trimester prenatal screening with a combined test improves the detection of Down syndrome (DS) cases with up to 90% accuracy and a 5% false positive rate^(1,2)^. In some circumstances, maternal serum screening and/or sonography can be false positive and this may result in performing unnecessary invasive procedures. Obstetricians use various methods to attain fetal cells for diagnosis, including chorionic villus sampling (CVS), amniocentesis (AC), and cordocentesis (CC). Detection of smaller CA other than structural and numeric CA can be performed due to recent improvements in medical genetics. Molecular DNA techniques allow detection of numerous single-gene disorders^(3)^. There is also a revolutionary development in prenatal screening that helps obstetricians screen an extensive scale of genetic disorders with a cell-free DNA technique.

Our aim was to investigate the indications, types of genetic techniques, fetal karyotype results, and pregnancy outcomes of women in a university hospital for performing invasive diagnostic procedures over a period of 7 years.

## MATERIALS AND METHODS

This is a retrospective cohort study of pregnant women who underwent genetic invasive procedures (AC, CVS or CC) between January 2010 and January 2017 at a university hospital, in the department of obstetrics and gynecology. The institutional ethics committee of our university hospital approved the study (approval number: 2017/12). A total of 2185 prenatal samples were included in the study. All patients signed an informed consent form following a genetic consultation and during which the risks, benefits, and limitations associated with screening and invasive tests were explained. We performed a sonographic examination of all patients to detect any morphologic abnormalities prior to invasive procedures. CVS, AC, and CC were performed between 11 and 14 weeks of gestation, from 15 weeks, and from 21 weeks, respectively. The same obstetricians performed all invasive procedures. Indications for prenatal diagnostic testing were: maternal age, abnormal results of aneuploidy screening (combined first-trimester or biochemical second-trimester screening for DS >1/300 or 1:150 for trisomy 13 or 18), abnormal structural findings in fetal ultrasound (including increased nuchal translucency ≥3.5 mm before the introduction of the first-trimester screening, major fetal abnormality, and soft ultrasound markers), parental translocation carrier status, parental carrier status of a known genetic disorder, previous child with aneuploidy or other genetic disorder, and maternal request. Demographic parameters regarding maternal characteristics such as age, gravidity, parity, and gestational age were recorded. Indications for prenatal invasive procedures, types of invasive procedures and genetic laboratory tests performed, and finally, any complications related to procedures were analyzed. The methods used for the purpose of prenatal diagnosis were conventional cytogenetic techniques (G-banding preparations), fluorescence in-situ hybridization (FISH), quantitative fluorescence polymerase chain reaction (QF-PCR), and molecular DNA techniques. FISH and QF-PCR were performed for rapid identification of trisomies (13, 18, and 21). We used G-banding for chromosomal karyotyping in patients at risk of aneuploidy, and DNA testing for specific mutations that cause disease in patients at risk of a genetic disorder.

The analysis of chromosomal aberrations was classified as numeric (autosomal trisomy -21, 18, 13, 17, 7-, monosomy, triploidy, and sex CA), structural (inversion, deletion, de novo marker, Robertsonian translocation, reciprocal translocation, chromosomal variant), and single gene disorders (fragile X syndrome, maple syrup disease, spinal muscular atrophy, congenital adrenal hyperplasia, thalassemia). Chromosomal mosaicism was also included in structural chromosomal aberrations.

We performed a standard procedure that consisted of an ultrasound-guided transabdominal approach using an 18-G needle for CVS and 20-G needle for AC. We discarded the first 1-2 mL of the AC specimen and dissected chorionic villi from maternal decidua carefully to avoid maternal cell contamination. The mean amount of amniotic fluid obtained using AC was 16-20 mL.

## RESULTS

There were 2185 invasive procedures, which consisted of 1853 AC, 326 CVS, and 6 CC over a period of seven years. Two thousand one hundred eighty procedures were performed in singleton pregnancies and 5 in twins. The performed invasive tests with regard to indications are shown in Table 1 and the summary of CA and genetic disorder rates is shown in Table 2. The most common indication for prenatal invasive procedures was abnormal results of aneuploidy screening for trisomy 21, followed by maternal age, and fetal structural abnormality. Fetal karyotype could not be revealed in 42 (2%) cases due to maternal contamination in 18 cases, inadequate sampling in 4 cases, and failure of cell culture in 27 cases. A second sampling procedure was performed and a normal karyotype was revealed in seven cases with a failure of cell culture at the first procedure. The genetic laboratory techniques performed in our cases for testing of fetal samples were: conventional cytogenetic analysis (n=1974, 90.5%), QF-PCR (n=163, 7.5%), FISH (n=4, 0.2%), and molecular DNA testing (n=38, 1.7%). There were 154 (7%) cases with chromosomal structural or numeric abnormalities and 15 (0.7%) cases with a genetic disorder. An analysis of cases with CA revealed 145 results with disease-causing CA and 9 with chromosomal variants. The summary of CA diagnosed in our study is shown in Table 3. The most common numerical CA was trisomy 21 (73/2185; 3.3%), the most common structural CA was reciprocal translocation (13/2185; 0.6%) and chromosomal inversion (13/2185; 0.6%). There were two cases of trisomy 21 fetal karyotype coexistence with Robertsonian translocation in one case and Klinefelter sex CA in another case. The outcomes of pregnancies associated with CA and genetic disorders are listed in Table 4. Finally, there were 2 pregnancy losses due to the invasive procedures (only in AC).

## DISCUSSION

CA of the fetus can result in numerous complications, including abnormal phenotype (1/150 of live births)^(3)^, miscarriage in the first trimester (50% of recognized miscarriages), and stillbirth in the second trimester (5% of stillbirths)^(4)^. Detection of CA in the prenatal period may help to decrease these complications. Currently, prenatal screening and/or diagnostic testing for aneuploidy is offered irrespective of age or risk^(5)^. In obstetric practice, an abnormal result of aneuploidy screening replaced maternal age (35 years or over) as the most common indication for invasive procedures. Besides, structural abnormalities comprise up to 12% of indications in most studies^(6,7)^. In our study, consistent with the literature, the most common indication for invasive procedures was an abnormal result of aneuploidy screening for trisomy 21, which was followed by maternal age and fetal structural abnormality, with rates of 69%, 11.25%, and 10.5%, respectively. These indications can vary, probably due to factors such as a difference between public health policies for screening CA, difficulties in accessing obstetric care, fear of adverse results related to the procedures, and absence of treatment after certain diagnoses. The rates of other indications were also compatible with the literature^(8,9)^. Our study demonstrated a CA rate up to 7% (154/2185)^(7,10,11)^. Another study in a Turkish population focused on the same issue and revealed a CA rate of 4.4%^(12)^. The medical literature reports culture media failure lower than 1.0%^(11,13,14,15)^ and we observed approximately the same rate (1.2%). The patients and their spouses accepted the second attempt in only 7 of 27 cases (6 CVS and one AC) and a new AC was performed, all of which revealed a normal karyotype.

In cases of CA incompatible with extra-uterine life, awareness of this fetal karyotype has allowed women and their spouses to make decisions about their pregnancies if they prefer a legal induced abortion. As a secondary benefit, the knowledge of fetal karyotype also helps to alleviate anxiety throughout the pregnancy related with abnormal screening results. We preferred AC over other invasive procedures due to their diagnostic reliability, ease of the procedure, late obtainment of results, and a relatively low fetal loss rate. A current estimate of invasive procedure-related loss in experienced hands for AC and CVS are 0.1% and 0.2%, respectively^(16)^. We performed CC in a limited number of circumstances due to concerns of a higher risk of miscarriage and late obtainment of results. In general, CVS is less preferred than AC, which leads to a slightly higher risk of miscarriage, and possible false-positive results due to confined placental mosaicism (usually requires confirmatory diagnosis with AC).

Cytogenetic analysis deals with viable cells obtained from CVS, AC or CC, whereas DNA molecular testing uses either viable or non-viable cells for analysis. There are many different laboratory techniques that can be used for testing fetal samples: karyotyping, FISH-interphase, chromosomal microarray analysis (CMA), and molecular DNA testing. Analysis of cultured fetal cells allows detection of CA larger than 5-10 Mb^(3)^. In addition, CMA enables better determination of minor CA and microdeletions by increasing the detection rate by about 2.9% as compared with conventional karyotyping^(17)^. CMA is used on very limited occasions in a restricted number of laboratories and is not accessible with cost-effective prices in Turkey. We do not use this method in our clinic. Neither CMA nor FISH examinations can detect non-disjunction and translocation aneuploidy. Balanced translocations are usually associated with normal phenotype and may have serious complications including recurrent miscarriage and an increased risk of having abnormal offspring^(3,18)^. In the present study, 16 translocations (14 reciprocal and 2 Robertsonian) were detected. The risk of a serious congenital anomaly in such translocations is expected as 3.7% for Robertsonian translocations, 6.1% for de novo reciprocal translocations, and 9.4% for inversions^(18)^. Triploidy is incompatible with life; very few fetuses have lived beyond 20 weeks of gestation in the literature. Early termination of pregnancy restricts complications and maternal mortality associated with triploidy^(19)^. There was only one triploidy in our study and we induced abortion after genetic counseling. A rapid, economic, and accurate diagnosis of common aneuploidies (trisomy 21, 18, 13) is available with QF-PCR. Unfortunately, some chromosome aberrations cannot be diagnosed using QF-PCR, as such, this technique cannot be solely used instead of conventional cytogenetic analysis^(20,21)^. In our study, we performed QF-PCR more commonly in comparison with FISH analysis for rapid identification of trisomy (13, 18 and 21) beyond 20 weeks of gestation.

Chromosomal mosaicism is defined as the existence of different cell types in a tissue with a rate of 0.25% for AC and 1% for CVS^(22,23)^. In our study, we diagnosed two cases of mosaicism following AC and abortion was induced in both after genetic counseling. This low rate of mosaicism in our study may have resulted from the high AC preference with regard to CVS. The skill and practice of the obstetrician plays an important role in performing invasive procedures^(14,15,24)^. In our study, we observed only 2 abortions (0.09%) associated with invasive procedures. Our obstetrics team is experienced with invasive procedures and the same physicians (authors of this study) always perform these procedures. This finding may have contributed to the very low procedure-related miscarriage rate as compared with the literature.

In summary, 4.7% of cases were detected as having chromosomal trisomy 13, 18 or 21 (102/2185), 2.4% (52/2185) of cases had other chromosomal rearrangements, and in 0.7% (15/2185) of cases, single gene disorders were present (Table 2).

In Turkey, pregnancies with genetic abnormalities can legally be terminated at up to 24 weeks of gestation. Patients and spouses were informed about the presence of a genetic abnormality and 49% (76/154) of our patients requested dilatation and curettage. Genetic testing was repeated after induced abortion and the same genetic results were confirmed in all cases.

In contrast, 23% (36/154) of the patients declined abortion and resumed their pregnancy. The reasons for this rejection included religious beliefs, very desired pregnancies, or twin pregnancies (no option for selective abortion). We could not perform confirmatory genetic testing for the postnatal follow-up of cases in which abortion was declined. These patients were all referred from other hospitals and all were lost to follow-up. Patients lost to follow-up accounted for 27% (42/154) of patients with a genetic disorder.

The strengths of this report are the chromosomal abnormality-single gene disorder diversity and performance of invasive methods by the same obstetric team. As far as we know, this is one of the largest and most comprehensive studies reported from the Southeast Anatolian region, which may give crucial clues for a specific Turkish women population regarding prenatal genetic diagnosis.

### Study Limitations

The retrospective nature and limited number of CC are limitations of our study.

## CONCLUSION

In conclusion, women face many problematic issues while undergoing invasive procedures, including the possibility that the fetus will have a CA or single-gene disorder, the risk of procedure-related miscarriage, and the consequences of a child with CA. Improved laboratory facilities, more accessible high-technology ultrasound equipment, and developed cytogenetic technology (detection of microdeletions and microduplications) contribute to increased prenatal detection of chromosome abnormalities. The rate of CA of newborns can be decreased through prenatal detection of abnormal karyotypes, which may result in reduced social trauma and financial load on both the parents and society due to disabled children. The ideal approach to pregnancies with detected CA should be tailored according to the individual choice of the couples regarding whether they decide for or against a child with a known CA.

## Figures and Tables

**Table 1:**
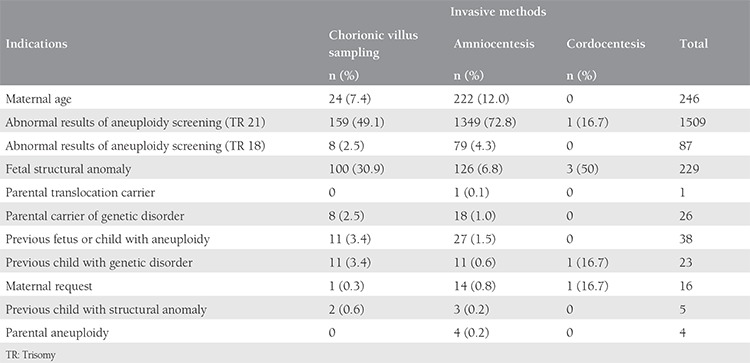
Invasive test methods with regard to the indications

**Table 2:**
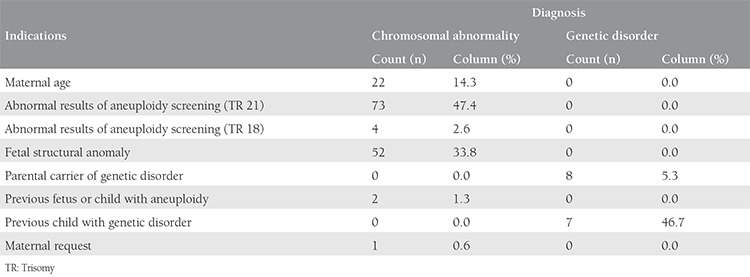
Summary of chromosomal abnormalities and genetic disorder rates

**Table 3:**
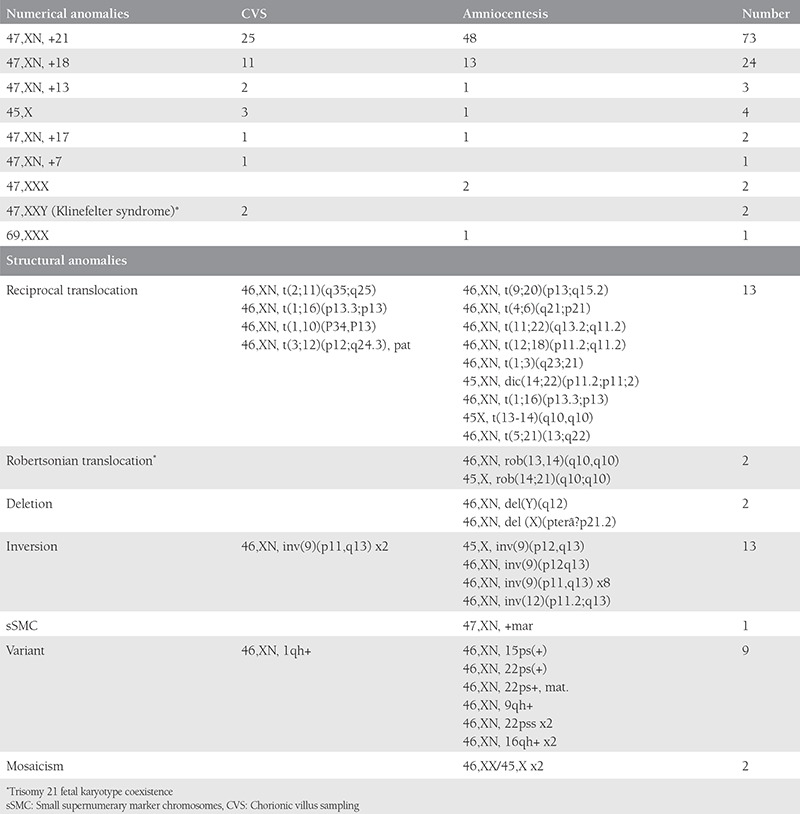
Summary of the chromosomal abnormalities diagnosed in our study

**Table 4:**
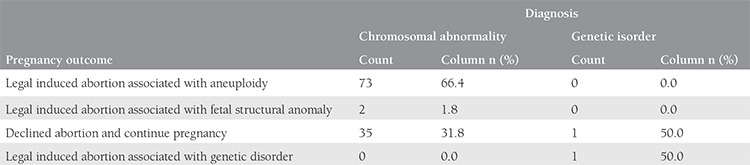
The outcomes of pregnancies associated with chromosomal abnormalities and genetic disorders
